# Metabolic and endocrine changes induced by cinnamon in women with polycystic ovarian syndrome: A pilot study

**DOI:** 10.22038/AJP.2023.23357

**Published:** 2024

**Authors:** Sepideh Peivandi, Sahar Heydari-latibari, Fatemeh Ghasemzadeh, Marzieh Zamaniyan, Adele Bahar, Hadi Majidi, Behnam Maleki

**Affiliations:** 1 *Sexual and Reproductive Health Research Center, Department of OB/GYN, IVF Ward, Sari Imam Khomeini Hospital, School of Medicine, Mazandaran University of Medical Sciences, Sari, Iran*; 2 *Department of OB/GYN, IVF Ward, Sari Imam Khomeini Hospital, Mazandaran University of Medical Sciences, Sari, Iran*; 3 *Diabetic Research Center, Department of OB/GYN, IVF Ward, Sari Imam Khomeini Hospital, School of Medicine, Mazandaran University of Medical Sciences, Sari, Iran*; 4 *Department of Endocrinology, Diabetic Research Center, Sari Imam Khomeini Hospital, School of Medicine, Mazandaran University of Medical Sciences, Sari, Iran*; 5 *Department of Radiology, Sari Imam Khomeini Hospital, School of Medicine, Mazandaran University of Medical Sciences, Sari, Iran*; 6 *IVF Ward, Sari Imam Khomeini Hospital, Mazandaran University of Medical Sciences, Sari, Iran*

**Keywords:** Cinnamon, Polycystic ovarian syndrome Menstruation, Insulin resistance, Lipids, Abdominal fat

## Abstract

**Objective:**

Most polycystic ovary syndrome (PCOS) patients have metabolic abnormalities in which insulin resistance (IR) plays a pivotal role. Cinnamon is a herbal medicine with insulinotropic properties. This pilot study aimed to evaluate the effects of cinnamon on ovarian volume, androgenic profile, and metabolic and anthropometric parameters in women with PCOS.

**Materials and Methods:**

A single-center, double-blind, randomized, placebo-controlled trial was carried out on 39 overweight / obese women with PCOS. For six months, subjects in the cinnamon (500 mg) (n=19) and placebo (n=20) groups were given three capsules daily. Before and after the intervention, the variables were assessed. The data was analyzed using the GraphPad Prism software.

**Results:**

After the intervention, the two intervention and control groups had significant differences in abdominal subcutaneous fat and ovarian volume, but they did not differ significantly in terms of body mass index (BMI). Also, after the intervention, no significant difference was observed between the two groups in terms of lipid profile and the concentration of androgenic biomarkers of insulin resistance.

**Conclusion:**

Cinnamon supplementation improves ovarian volume and subcutaneous abdominal fat but has no effect on anthropometric parameters, lipid profile, insulin resistance, or androgen hormones.

## Introduction

Polycystic ovary syndrome (PCOS) is the most prevalent endocrine disorder in women of reproductive age (Kataoka et al., 2019). PCOS has adverse reproductive, metabolic, and psychosocial consequences, resulting in a considerable decrease in health-related quality of life (de et al., 2019; Moghadam et al., 2018). Diagnostic criteria for this syndrome involve three characteristics: oligo- anovulation, clinical or biochemical hyperandrogenism, and polycystic ovary presentation on ultrasound imaging (Zawadzski, 1992; ESHRE and Group, 2004; Azziz et al., 2006; Dewailly et al., 2011). 

According to a systematic review and meta-analysis, the prevalence of PCOS in women of reproductive age ranges from 5% to 18% (Ding et al., 2017). This syndrome's symptoms include hirsutism, acne, hair loss, menstrual irregularity, and infertility, as well as reproductive and endocrine disorders. PCOS also has an adverse effect on metabolic health, raising the risk of cardiovascular disease and diabetes (Fauser et al., 2012; Sirmans and Pate, 2014). Genetic and epidemiological studies indicate an interaction between obesity and PCOS. The metabolic effects of obesity on PCOS pathogenesis are mediated by insulin resistance and adipokine synthesis in subcutaneous and visceral fat (Barber et al., 2019; Kim et al., 2019).

Recently, the role of hyperinsulinemia in abnormal androgen production, as well as irregular menstrual cycles and follicogenesis, which are the hallmarks of PCOS, has been reported (Nestler et al., 1998; Azziz, 2003). It seems that early detection and treatment of insulin resistance can prevent metabolic pathogenesis and reproductive endocrinopathy (Kim et al., 2019). In this regard, various glucose-lowering medications, including insulin sensitizers, have been introduced and prescribed, although there are concerns about the side effects of these chemical agents (Ashkar et al., 2019; Hayward et al, 2019; Sá., 2019).

Herbal medicines that increase steroidogenic response and estrogen receptor protein expression, and regulate glucose and lipid metabolism through antioxidant and anti-inflammatory compounds, are appropriate for use as a complement to PCOS treatment (Abasian et al., 2018; Sharifi-Rad et al., 2021). Cinnamomum verum is one of the natural products that have been studied for its ability to treat gynecological, hormonal, and metabolic diseases (Borzoei et al., 2018; Deyno et al., 2019; Shirzad et al 2021; Xu et al., 2020).

*In vivo* and *in vitro* studies have shown that cinnamaldehyde, the active component of cinnamon, has insulinotropic properties, including positive effects on insulin release and sensitivity. It also regulates protein-tyrosine phosphatase 1B (PTP1B) and insulin receptor kinase (IRK) (Wang et al., 2007; Ulbricht et al., 2011; Khodaeifar et al., 2019; Kumar et al., 2019). Aside from glucose metabolism, clinical studies have demonstrated that cinnamon supplementation improves lipid profiles and anthropometric parameters in patients with type 2 diabetes mellitus (T2DM) and obesity (Vallianou et al., 2014; Yazdanpanah et al., 2020). 

Although several studies have been conducted on the efficacy of cinnamon in controlling the metabolic parameters of PCOS (Heydarpour et al., 2020), few clinical trials have been conducted on the effects of cinnamon on other parameters of this syndrome, such as ovarian volume, lipid profile, androgenic biomarkers, and insulin resistance indices. The goal of this study was to assess the effects of cinnamon on ovarian volume, and androgenic hormonal profile, as well as metabolic and anthropometric parameters in overweight and obese women with PCOS.

## Materials and Methods

**Study design and participants**

This single-center, double-blind, randomized, placebo-controlled trial was conducted on PCOS patients referred to Imam Khomeini Hospital's gynecologic clinic in Sari, Iran. (IRCT code: 201706162825N2 and code of ethics IR.MAZUMS.REC.95-2061) PCOS was defined as having at least two of the Rotterdam criteria: oligo- anovation (oligomenorrhea or amenorrhea), clinical or biochemical evidence of hyperandrogenism, and ultrasound imaging evidence of a polycystic ovary (ESHRE and Group, 2004).

The inclusion criteria included the patient's willingness to participate in the research, BMI 25-35 kg/m2, no pregnancy or breastfeeding, no current fertility treatment, and no use of hormonal or antidiabetic drugs in the last three months. Exclusion criteria, amenorrhea, hypersensitivity to cinnamon and other herbal medicines, corticosteroids, antihypertensive drugs, anti-prostaglandins, aspirin and other anticoagulant drugs; convulsions; Cardiovascular or brain diseases; or liver, kidney or thyroid disorders.

**Sample size**

For sample size, we considered a total of 41 individuals. According to Whitehead et al.'s study, the needed pilot trial sample size per treatment arm for an 80% powered main trial with a small standardized effect size is 20 (Whitehead et al., 2016). The participants were chosen from PCOS patients referred to the gynecologic clinic as a convenience sample method.

**Randomization**

Participants were enrolled and determined by a midwifery staff member at a women's clinic who was not a member of the study team, during 6 months.

The allocation sequence was produced using a randomized block design with a block size of four, resulting in six balanced combinations with two A (intervention) and two B (control) as variables (AABB, ABAB, ABBA, BAAB, BABA, and BBAA). The medications were labeled A and B, which were indistinguishable from one another. The pharmacist had access to the codes while the researchers were unaware.

**Intervention**

The patients were separated into two groups: those who received the intervention (cinnamon) and those who received the placebo (control). Based on Kort and Lobofindings, we used a daily dose of 1.5 g cinnamon medication, which was administered as 500 mg capsules three times a day for six months (Kort and Lobo, 2014). Similarly, placebo capsules containing starch were given to control subjects. These capsules were prepared manually with identical appearance at the Pharmacognosy laboratory at Mazandaran University of Medical Sciences' School of Pharmacy.

Throughout the trial, participants were asked to continue their typical physical activity and follow a diet containing carbohydrates at 50%, protein at 15%–20%, and fat at less than 30% throughout the study (Foster et al., 2003; Yancy et al 2004). We had six regular monthly phone follow-ups to ensure that the research medicines were being used properly and that the advised diet and physical activity were being followed.

**Medication**

Consumable capsules had a plastic cover to cover the contents inside in terms of color and taste. This coating was digestible. The content inside for the intervention group contained cinnamon. For the control group, it contained starch. It was completely similar in taste and appearance. And it was made in the Faculty of Pharmacy, Faculty of Medical Sciences, Mazandaran, Iran.

**Outcomes**

We performed two clinical observations on individuals, one before and one after the operation. Abdominal ultrasonography (Madison WS80A) was used to measure ovarian volume in the early follicular phase, 3–7 days after menstruation. Furthermore, the thickness of subcutaneous abdominal fat at the sub-umbilical surface was assessed. All imaging work-ups were done at Imam Hospital's imaging center by our study team radiologist. 

Serum insulin, triglyceride (TG), high-density lipoprotein (HDL), total testosterone, and dehydroepiandrosterone sulfate (DHEA-S) were all measured in 5 ml peripheral venous blood samples.

Insulin resistance indices, such as the Homeostasis model assessment of insulin resistance (HOMA-IR) and the Quantitative insulin sensitivity check index (QUICK-I), were calculated as follows:

HOMA-IR=fasting insulin (μU/ml)×fasting glucose (mmol/L) /22.5

QUICK-I=1/Log[fasting insulin concentration (μU/mL)]×Log[(fasting blood sugar (mg/dL)]

Anthropometric parameters such as height and weight (to calculate BMI), as well as hip and waist circumference, were also measured. Furthermore, demographic data and menstrual history, including the last menstruation date, were recorded.

**Statistical analysis**

Statistical analysis was performed using GraphPad Prism version 8.4.2 (GraphPad Software, Inc., San Diego, CA). The D'Agostino-Pearson normality test was performed to determine the normality of the distribution of the data. Statistical significance for parametric and non-parametric variables was determined using the Student T test or the Mann-Whitney U test, respectively. The statistical significance level was set at 0.05. Data is presented as the mean±standard deviation.

## Results

While forty-one people participated in the trial, 39 of them completed it (cinnamon group: 19 and placebo group: 20). At the beginning of the study, there was no significant difference between the two groups in terms of the demographic, anthropometric, subcutaneous abdominal fat, ovarian volume, lipid profile, androgenic biomarker concentration or insulin resistance index.

**Figure 1:**
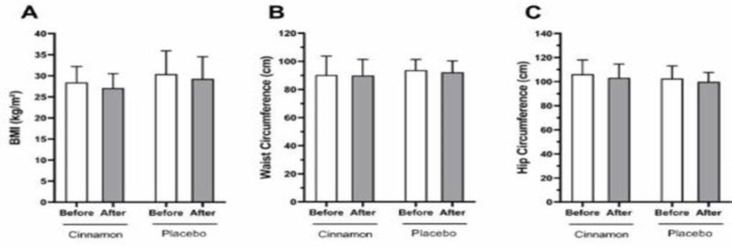
Comparison of anthropometric parameters before and after intervention in the cinnamon and placebo groups. The data is presented as the mean and standard deviation.

body mass index (BMI)

**Figure 2:**
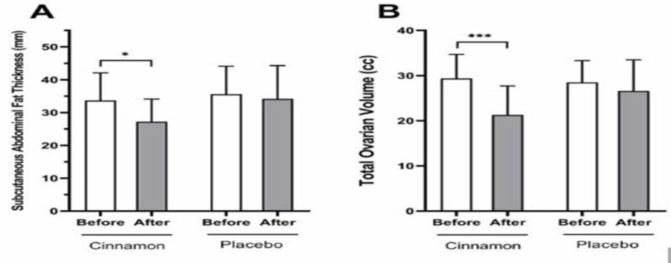
Comparison of subcutaneous abdominal fat and ovarian volume before and after intervention in the cinnamon and placebo groups. The data is presented as the mean and standard deviation. * shows a p <0.05, *** shows a p <0.001.

**Anthropometric parameters**

There was no significant difference in BMI, waist circumference or hip circumference before and after intervention in the cinnamon and placebo groups (shown in Figure 1). 

**Subcutaneous abdominal fat and ovarian volume**

As shown in Figure 2-A, subcutaneous abdominal fat thickness decreased during the study in the cinnamon group (p=0.01), but did not significantly change in the placebo group (p=0.63). After intervention, total ovarian volume significantly decreased in the cinnamon group (p=0.0002) (shown in Figure 2-B), nevertheless, no significant decrease was seen in the placebo group.

**Lipid profile and androgenic biomarkers concentration**

**Figure 3:**
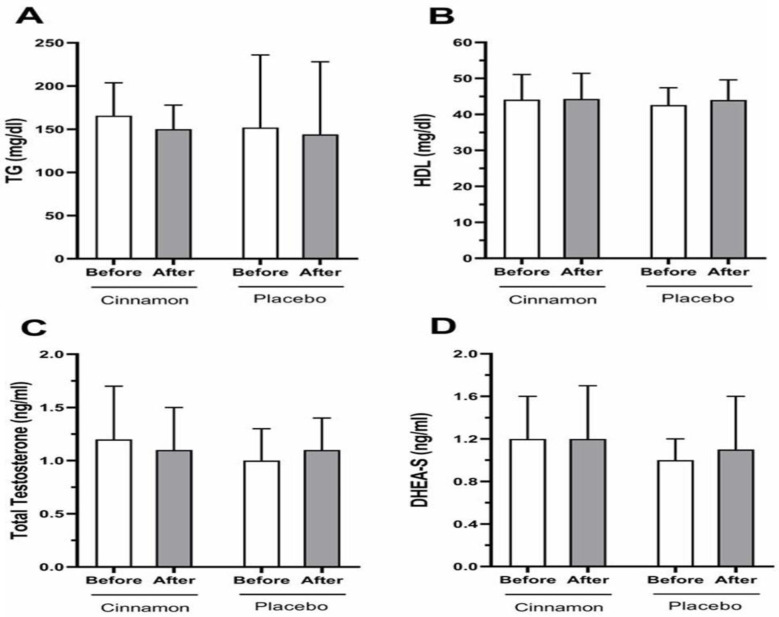
Comparison of the cinnamon and placebo groups lipid profiles (A, B) and androgenic biomarker (C, D) concentrations before and after intervention. The data is presented as the mean and standard deviation. Triglyceride (TG), high-density lipoprotein (HDL), dehydroepiandrosterone sulfate (DHEA-S)

In Figure 3, the before and after intervention concentration of serum lipid (TG and HDL) and androgenic biomarkers (total testosterone and DHEA-S) is demonstrated. No significant changes were seen in the concentration of TG or HDL in the cinnamon and placebo groups (shown in Figure 3A and 3-B). In relation to androgenic biomarkers, there was no significant difference between before and after intervention concentrations of total testosterone and DHEA-S in groups (Figure 3-C and 3-D).

**Insulin resistance indices**

**Figure 4:**
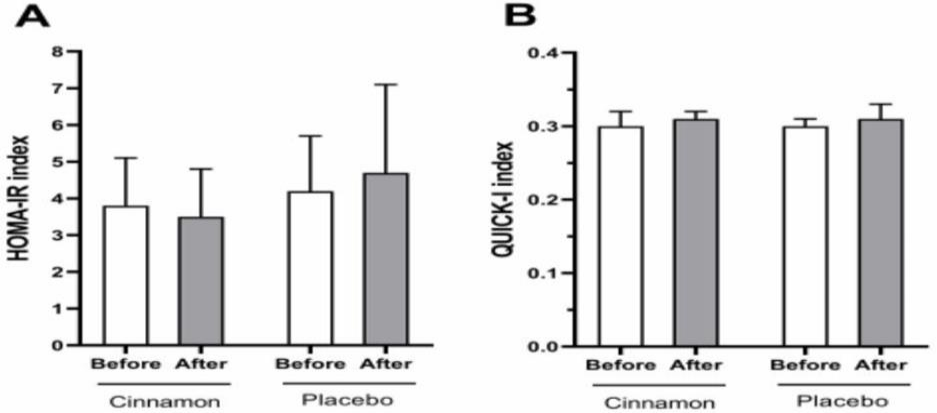
Comparison of insulin resistance indices in the cinnamon and placebo groups before and after intervention. The data is presented as the mean and standard deviation. Homeostasis model assessment of insulin resistance (HOMA-IR), Quantitative insulin sensitivity check index (QUICK-I)

The before and after intervention score of insulin resistance indices (HOMA-IR index and QUICK-I index) are demonstrated in Figure 4. Both groups had similar outcomes after intervention in terms of the HOMA-IR index and the QUICK-I index (shown in Figure 4). 

## Discussion

The purpose of this trial was to investigate the effects of cinnamon on metabolic and ovarian-related parameters in women with PCOS.

To the best of our knowledge, there is no scientific evidence for the effect of cinnamon on ovarian volume. Sipahi et al. (Sipahi et al., 2019) examined the predictive power of ovarian volume in determining metabolic syndrome in PCOS. Their study sample consisted of 96 women with PCOS who were referred for gynecological examinations and had their ovarian volume evaluated using Doppler and other ultrasonography methods. The findings showed that with increasing ovarian volume, the risk of developing metabolic syndrome increases. In our study, a significant reduction in total ovarian volume after cinnamon administration was observed. According to this finding, the mechanism of change in ovarian volume by cinnamon may be related to the effects of this substance on glucose and lipid metabolism.

We observed no significant changes in TG or HDL values, as well as HOMA-IR and QUICK-I index as insulin resistance indices. This finding is in line with the American Diabetes Association's declaration in its 2017 Diabetes Treatment Standards Report that there is insufficient evidence to support the use of herbs and spices, such as cinnamon, to treat diabetes (Association, 2017).

According to previously reported preclinical studies, cinnamon decreases IR through enhancing phosphatidylinositol 3-kinase activity in the insulin signaling pathway. This phenomenon leads to an increase in glucose absorption (Vallianou et al., 2014). Cinnamon's procyanides and catechins are primarily responsible for these pharmacological actions (Wang et al., 2007). Insulin plays a provital role in lipid metabolism via the same mechanism, hence a rise in serum insulin decreases lipid levels (Wang et al., 2007). Moreover, other preclinical studies have shown that the dietary fiber in cinnamon reduces intestinal lipid absorption. Also, many vitamins and antioxidant compounds in this plant increase lipid metabolism (Ranasinghe et al., 2013).

In the current investigation, total testosterone and DHEA-S indices did not change following the consumption of cinnamon. These findings are consistent with the lack of change in the lipid and glycemic profiles in the patients investigated. Recent research has addressed the pivotal role of the two main factors, hyperandrogenemia (HA) and IR, and their interaction in the pathophysiology of PCOS symptoms. According to these studies, the underlying mechanisms of disorders of lipid metabolism and reproductive failure are linked to HA and IR (Wang et al., 2019; Sanchez-Garrido and Tena-Sempere, 2020).

In the present study, cinnamon supplementation showed no effect on reducing anthropometric parameters such as BMI, waist circumference, and hip circumference. Moreover, subcutaneous abdominal fat thickness was reduced, which is a positive point for controlling IR by cinnamon supplementation (Kelley et al., 2000). This study also reported the same results as the current study. A systematic review and meta-analysis of 21 clinical trials including 1480 adult participants found that cinnamon supplements lower BMI and weight by an average of 0.4 kg/m^2^ and 0.92 kg, respectively, but do not induce significant changes in waist circumference or body fat mass (Heydarpour et al., 2020). Furthermore, according to the conclusions of a systematic review and meta-analysis of 18 randomized clinical trials, cinnamon supplements did not induce significant changes in anthropometric indicators such as body weight, BMI, or abdominal circumference in type 2 diabetes patients (Namazi et al., 2019).

The main limitation of this study is the small sample size. We designed this research as a pilot study based on other researchers' reports (Foster et al., 2003) on the high probability of individual non-participation and the high rate of lost to follow-up in such trials, for reasons such as the need for periodic attendance, the long geographical distance between the residence and the clinic, and the difficult adherence to trial drugs as well as the recommended diet. Furthermore, several factors that influence body composition, metabolic and hormonal characteristics, such as genetics, individual metabolism rate, and nutrition, were uncontrollable. In this study, a fixed dose of cinnamon was used, so the results may not be generalized to other doses.

Despite these limitations, the strength of our study was addressing the less studied topics of cinnamon's effects on ovarian volume (as a surrogate measure for response to treatment of metabolic abnormality) (Sipahi et al., 2019) and subcutaneous abdominal fat thickness (as a surrogate measure of insulin resistance) (Kelley et al., 2000) in PCOS women.

In conclusion, findings from the current study indicate that administration of 1.5 g of cinnamon supplementation for 6 months can improve ovarian volume and subcutaneous abdominal fat thickness but had no effect on BMI and other anthropometric parameters, lipid profile, or insulin resistance indices in women with PCOS. Patients with PCOS who are overweight or obese may benefit from adding cinnamon extracts to their diets as a potential treatment for PCOS metabolic risk factors. Nevertheless, more studies with a larger sample size and the use of various doses of cinnamon are suggested. The finding will be useful for making a decision about introducing cinnamon supplementation as an approved complementary treatment for the improvement of hormonal and metabolic abnormalities in PCOS individuals.

## Conflicts of interest

The authors have declared that there is no conflict of interest.
